# Prefrontal Cortex Activation and Young Driver Behaviour: A fNIRS Study

**DOI:** 10.1371/journal.pone.0156512

**Published:** 2016-05-26

**Authors:** Hannah J. Foy, Patrick Runham, Peter Chapman

**Affiliations:** School of Psychology, University of Nottingham, Nottingham, United Kingdom; Tokai University, JAPAN

## Abstract

Road traffic accidents consistently show a significant over-representation for young, novice and particularly male drivers. This research examines the prefrontal cortex activation of young drivers and the changes in activation associated with manipulations of mental workload and inhibitory control. It also considers the explanation that a lack of prefrontal cortex maturation is a contributing factor to the higher accident risk in this young driver population. The prefrontal cortex is associated with a number of factors including mental workload and inhibitory control, both of which are also related to road traffic accidents. This experiment used functional near infrared spectroscopy to measure prefrontal cortex activity during five simulated driving tasks: one following task and four overtaking tasks at varying traffic densities which aimed to dissociate workload and inhibitory control. Age, experience and gender were controlled for throughout the experiment. The results showed that younger drivers had reduced prefrontal cortex activity compared to older drivers. When both mental workload and inhibitory control increased prefrontal cortex activity also increased, however when inhibitory control alone increased there were no changes in activity. Along with an increase in activity during overtaking manoeuvres, these results suggest that prefrontal cortex activation is more indicative of workload in the current task. There were no differences in the number of overtakes completed by younger and older drivers but males overtook significantly more than females. We conclude that prefrontal cortex activity is associated with the mental workload required for overtaking. We additionally suggest that the reduced activation in younger drivers may be related to a lack of prefrontal maturation which could contribute to the increased crash risk seen in this population.

## Introduction

Young drivers (aged 16 to 24) consistently account for the greatest proportion of accidents and fatalities on the roads [1–3]. Within this population there are different age groups; as defined by the World Health Organisation [4] adolescents are aged 16 to 19 whereas young people are defined as anyone 24 and under. More in depth examination of the young driver population has shown that collision reduction with age is also evident when comparing adolescents to those aged 20 and over [3] and when looking at year on year changes in accident rates [3,5]. More specifically, Maycock (2001) [6] modelled results of an accident liability survey [7] of around 13,500 drivers. Calculations from this model demonstrate a 10% reduction in accident rates between ages 18/19 and 21/22 when the effects of driving experience are accounted for. These statistics suggest an underlying problem that decreases with driver age.

A commonality among young drivers is their lack of driving experience. As with age, increases in experience are associated with a decrease in both collision and fatality rates [8,9]. Young driver crashes are often attributed to inexperience and may in part explain the higher collision and fatality rate of these drivers. However, young novice accident rates are double that of older novices in the first months of unsupervised driving and consistently higher than older novices for the first 24 months [10]. Therefore, a lack of experience cannot be the sole explanation.

Gender is also an influential factor with nearly three times as many males to females involved in road traffic injuries and fatalities each year [11]. Aggressive, and high risk road traffic collisions which result in fatalities are particularly more common in male than female drivers [12–14]. Males also self-report taking more driving related risks including; exceeding the speed limit, tailgating and overtaking when illegal or unnecessary [15–17].

As well as risk taking being prominent in males [18], adolescents are generally seen to be less risk averse than adults [19,20] and the common crash types of these young novice drivers also supports a theory of increased risk taking, for example; speeding, overtaking, rear end shunts and losing control of the vehicle are particularly common in young driver accidents [6,21,22].

These common collision types and the inability of experience factors to entirely explain young driver collisions emphasises the importance of other common and possibly unidentified causes. The current research analyses the prefrontal cortex (PFC) activity of young drivers based on the suggestion that increased crash risk in this population may be related to the maturational process of the PFC [23] and in accordance with neuroimaging evidence showing associations between reduced activation and maturation [24,25].

Development in the brain occurs in a back to front pattern, with the PFC being the last area of the brain to fully develop [26]. This is a process which is not complete until around 25 years of age in typically developing adults, [27,28] which is also the age that shows a significant decrease in road casualty risk [29]. More specifically, research has shown linear increases in PFC white matter with age, a process which begins in early childhood and continues through adolescence until the mid-twenties [27,30]. These structural changes have also been found to correlate with increases in PFC activity with age as demonstrated by Kwon, Reiss and Menon (2002) [31] who found linear increases in activity in both the right and left hemispheres of the PFC from ages seven to 22 during a working memory task. The PFC is linked to a number of factors including memory, emotion and decision making. This research examines in more depth the role of the PFC in inhibitory control and mental workload; both of which have been linked to accident risk.

Inhibitory control is the ability to weigh up consequences and suppress impulsive and inappropriate behaviours; all of which are believed to be heavily dependent on the PFC [32,33]. As already discussed, young novice and particularly male accident types are typically representative of high risk and poor inhibitory control. Neuroimaging methods have also shown correlations between younger age groups, increased risk taking behaviour and reduced prefrontal activity [34,35]. Similarly, patients with damage to the PFC make higher risk decisions [36–38]. Stimulation of specific PFC regions (e.g. Dorsolateral PFC) has also been reported as leading to safer driving behaviours in a simulator such as fewer speeding errors and increased headway [39]. Thus a lack of PFC activity could be associated with the high levels of risk taking seen in the young driver population.

Although there is no universal definition of mental workload, it is typically seen as the amount of operator resources that is required to meet task demands [40]. Mental workload related problems have been described as being responsible for the majority of road traffic accidents [41] with both high and low levels causing insufficient perception and attention [42–44] which in turn leads to driver error; a factor that is accountable for up to 90% of accidents [45,46]. High levels of mental workload can also be linked to young novice accidents in that for inexperienced drivers operating a vehicle is not an automatic task. In contrast, experienced drivers have acquired more effective automation through practice. Therefore, driving induces a higher level of mental workload for novices compared to more experienced drivers [47]. As with inhibitory control, neuroimaging techniques have demonstrated increases in PFC activation with increases in mental workload [48]. After a certain threshold further increases in workload lead to poorer performance and decreases in PFC activity [49]. This threshold can further explain young novice accidents in that reduced activation may be associated with reduced capacity [34] and also a significantly lower threshold, meaning that errors occur more frequently.

The current research examined the PFC activation of this at risk young driver population during driving tasks which were designed to manipulate mental workload and inhibitory control levels. This was done with simulated following and overtaking driving tasks and using functional near infrared spectroscopy (fNIRS) to measure PFC activity.

Brain activity during driving has previously been measured using a range of techniques including functional magnetic resonance imaging (fMRI) and positron emission tomography (PET). However, these methods typically require participants to be in a supine position. Along with the high sensitivity for motion artefacts these techniques pose issues when attempting to create realistic driving scenarios. In contrast fNIRS is an extremely portable technique which can be used in both simulated and real world driving; is more robust to issues such as motion artefacts [50] and has a higher temporal resolution when compared to a number of other techniques including fMRI and PET. fNIRS is a functional neuroimaging technique which has been used extensively to record changes in brain activation as measured by changes in the concentration of oxygenated and deoxygenated haemoglobin. This is based on their different absorption spectra of near infrared light and with respect to a resting baseline condition. Evidence of an association between haemoglobin levels and white matter [51] suggests that the developmental brain changes discussed earlier may also be evident in changes in haemodynamic concentration as measured by fNIRS. More specifically, fNIRS has been used to show that increases in prefrontal activation are associated with increases in development [24,52] such associations have also been found using fMRI [25].

Research using this technique provides further support for the activation of the PFC as a result of mental workload [53] and inhibitory control [54] manipulations. However, much of this research does not use naturalistic tasks and none has focused on differences in PFC activity specifically within the young novice driver category which may be an additional accident risk factor for this population.

Accordingly, a number of predictions were made: with respect to PFC activity; younger drivers will have less PFC activation than older drivers and inhibitory control and mental workload increases will be associated with increases in PFC activity. With respect to driver behaviour young drivers will overtake more than older drivers and male drivers will overtake more than female drivers, consistent with the increased risks seen in young male populations. Here we report our findings that PFC activation differed as a result of manipulations of driver age and task demands.

## Methods

### Participants

Although psychological research has been criticised for relying on undergraduate participants [55], this group has considerable advantages when looking at the young driver population and age and experience effects within it. Typically undergraduate students fall into demographic groups that span the ages 18 to 22 and include a wide variety of driving experience: while many drive frequently some will have done little or no driving since passing their practical test. This makes it possible to separate out differential influences of age and experience within an experimental design. A total of 32 participants from the University of Nottingham participated in the current study. Participants were split into eight groups of four based on their age (younger or older), experience (novice or experienced) and gender (male or female). A younger driver within this population was an adolescent aged 18 or 19 (*M* = 19.19 *SD* = .43) and older drivers were aged 21 to 22 (*M* = 21.42 *SD* = .30). Novice drivers were represented by those who had driven under 5,000 miles (*M* = 1631.31 *SD* = 1655.52) since passing their test and experienced drivers had driven over 10,000 miles *(M* = 23125 *SD* = 32573.64). All participants held a full UK driving licence.

The experimental procedure is in accordance with the principles set out in the Declaration of Helsinki and was reviewed and approved by the School of Psychology ethics committee at the University of Nottingham. All participants received a full explanation of the procedures and provided written informed consent for participation in this study.

## Materials

### Driving Simulator

**Fig 1:**
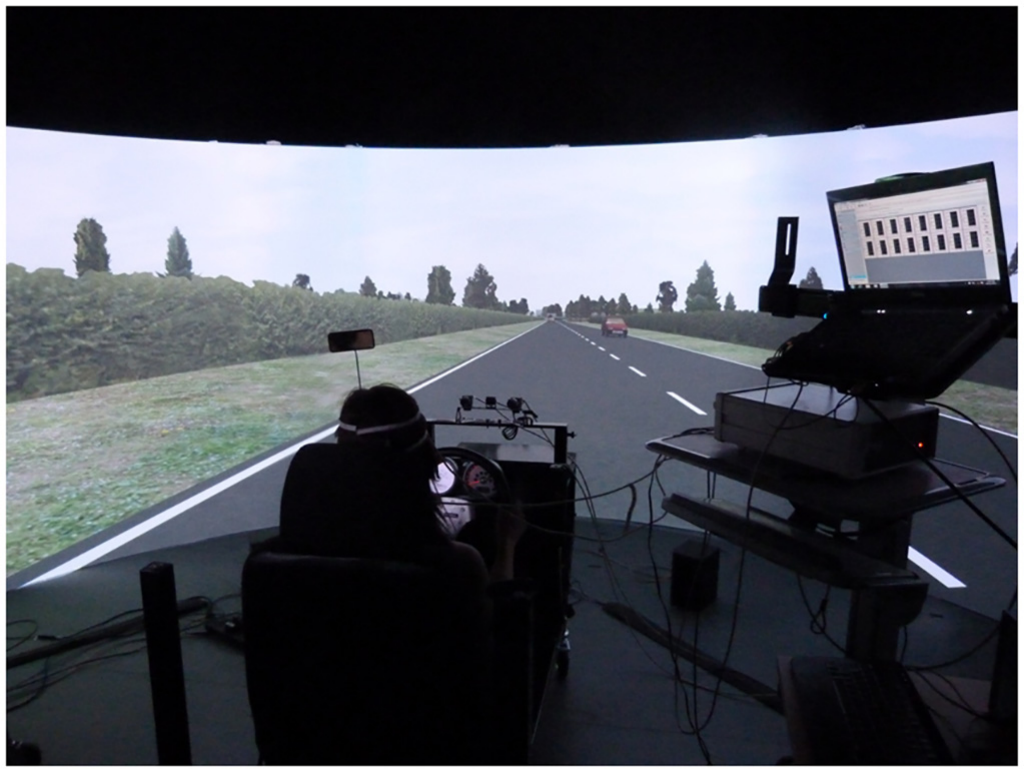
NITES 2 driving simulator and fNIRS apparatus. Photograph of the experimental setup. The participant is driving along the single carriageway road used in this experiment. The fNIRS computer is shown on the right; during the experiment this was positioned behind the participant out of their field of view.

The experiment took place in the Nottingham Integrated Transport and Environment Simulation (NITES) facility’s fixed base, mid-level fidelity driving simulator (NITES 2) (Fig 1). This comprises of a car rig (driver’s seat, steering wheel, gear stick, accelerator, brake and clutch) positioned facing the centre of a 180 degree circular projection screen (5 metre diameter) subtending 180 degrees of the visual field and a rear view mirror allowing participants to see the rear display screen (36 inch LCD television). The driving scenarios are formed on the screen using three projectors. From the driver’s perspective they see the road ahead but no part of the simulated car that they are operating, although this car occupies the same area of space on the road that a real car would (with the participant seated on the right hand side).

### Driving Scenarios

XPI (XPI Simulation, London, UK) driving simulation software was used to create five driving scenarios which were presented to participants in a random order. All scenarios took place along a 60mph stretch of single carriageway (Fig 1) and each lasted approximately three minutes. Four of the five scenarios were overtaking tasks at different traffic densities. For the purposes of this experiment the highest (High) traffic density was considered to have 100% oncoming traffic, with approximately 83 passing cars during the scenario. In relation to this the high medium condition had 83%, low medium had 66% and low had 47% oncoming traffic. The traffic on the drivers’ side of the carriageway was kept constant in all scenarios in order to allow equal opportunities to overtake. These vehicles were also programmed to travel at a relative speed which was 10mph slower than that of the participant; similarly this was to create opportunities to overtake. The fifth scenario was a following task along the same stretch of carriageway. These scenarios were designed to manipulate and dissociate mental workload and inhibitory control. More specifically, the following task was designed to elicit low levels of both mental workload and inhibitory control compared to the overtaking tasks which were designed to require higher levels of both. The increases in traffic density were expected to dissociate workload and inhibitory control. As the nature of the task remained the same increases in density were expected to maintain mental workload, but create less opportunity to overtake thus requiring a greater suppression of unsafe and impulsive behaviours and therefore greater levels of inhibitory control with increases in traffic density.

### fNIRS device

**Fig 2:**
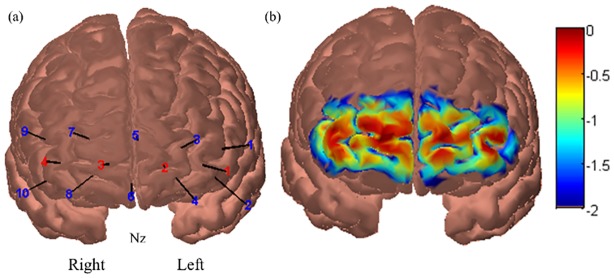
fNIRS probe placement. (a) Positioning of the 4 light sources (red) and 10 detectors (blue) with references to the nasion. (b) Sensitivity profile of the fNIRS probe used in this experiment projected onto a digital brain atlas based on the “Colin27” atlas [57] commonly used in MRI studies. The colour scale depicts the sensitivity logarithmically. Both images were created using AtlasViewerGUI [58].

A BIOPAC 100A (BIOPAC Systems Inc, USA) continuous wave fNIRS device was used to measure PFC activity. This particular device records at a frequency of 2Hz and consists of a sensor pad (180x60x8mm) fitted with 4 LED light sources with an inter-optode distance of 25mm that emit near infrared light at 730nm and 850nm wavelengths, which are absorbed primarily by deoxygenated and oxygenated haemoglobin respectively. The sensor pad also houses 10 light detectors (2.3mmx2.3mm silicon photodiode with integrated trans-impedance preamp), creating 16 recording channels. The software used for recording the fNIRS data is the Cognitive Optical Brain Imaging (COBI) Studio (fNIR Devices, Potomac, MD, USA) and HomER2 software was used for the pre-processing of fNIRS data [56]. The fNIRS device was placed behind the participant in the driving simulator (Fig 1) and setup was measured with the centre of the device in line with the nasion (Fig 2). Care was taken to avoid hair from the eyebrows or side of the head interfering with detectors and sources.

### Questionnaires

Participants completed a brief driving demographic questionnaire prior to the experiment. This questionnaire was used to record participants’ age, gender and driving experience. Experience was measured by the number of miles driven since passing their driving test. Participants were given tables of average miles driven by different age and gender groups as well as distances between major UK cities in order to aid them in their annual and total mileage estimates (for demographic data see S1 appendix). An extended NASA-TLX workload questionnaire was completed after each scenario. This is a self-assessed measure based on six 20 point scales with 0 being “Very Low” and 20 “Very High.” The scales are: Mental Demand, Physical Demand, Temporal Demand, Performance, Effort and Frustration. Mental Demand was the scale of interest for mental workload and asked participants “How mentally demanding was the situation?” A seventh scale of Inhibitory Control was also added with the wording “How hard did you have to try to prevent yourself from performing dangerous manoeuvres when inappropriate?” This questionnaire was used to subjectively assess mental workload and inhibitory control levels.

### Procedure

Following a five minute practice along roads not used in the experiment participants drove each of the five scenarios in a random order. All drivers were told prior to each scenario whether they were completing a following or overtaking task. For following tasks participants were instructed to simply follow the vehicle ahead, while for overtaking tasks participants were told that they could overtake if it was safe to do so. After each drive the participant completed the extended NASA-TLX questionnaire. PFC activity was recorded using fNIRS throughout each scenario.

### fNIRS data pre-processing

Raw fNIRS data (16 channels x 2 wavelengths) was pre-processed using HomER2 software, a graphical interface programme executed in Matlab (Mathworks Inc., Sherborn, MA) for visualisation of optical data. The function hmrPruneChannels was used to identify any ‘poor quality’ channels in which the signal was too weak, too strong or their standard deviation too great. For 15 participants channels 5, 6, 9 and 10 were identified as poor quality channels, these were thus removed from pre-processing and from further analysis for these participants. In order to address any artefacts caused by participant motion the function hmrMotionCorrectWavelet was used. For this wavelet transform a probability threshold (α) of 0.1 was selected as adopted in previous studies [59–61]. This process reduces artefacts in up to 93% of cases [59] and therefore a further motion detection process was conducted using the hmrMotionArtifact algorithm. Here motion artefacts were identified as a signal change (optical density units) greater than an amplitude of 0.3 over half a second, any such time-points were marked for one second and removed as a motion artefact; no additional artefacts were identified. A low-pass filter (3^rd^ order Butterworth filter) of 0.5Hz was applied in order to reduce high-frequency instrument noise and physiological noise such as fast cardiac oscillations (e.g. heartbeat 1~1.5Hz). This filter band has also been adopted in previous fNIRS research [62,63]. HomER outputs changes in oxygenated haemoglobin (ΔOxyHb), deoxygenated haemoglobin (ΔDeoxyHb) and total haemoglobin (ΔTotalHb = ΔOxyHb + ΔDeoxyHb). The results of this experiment focus primarily on ΔDeoxyHb as this measure tends to be most highly correlated with other neuroimaging measures such as the fMRI measured BOLD response both theoretically and in practice [64–66]. It should be noted that strong correlations with both ΔOxyHb and ΔTotalHb have also been found [67]. All fNIRS results are reported in micromoles (μM).

### Data Analysis

Analyses were conducted to determine whether inhibitory control and mental workload were successfully manipulated during these scenarios; whether driver age, experience and gender influenced risk taking manoeuvres (i.e. overtaking) and whether there were differences in PFC activity between the different tasks and the different driver categories (e.g. age). 2 (age) x 2 (experience) x 2 (gender) x 2 (overtake vs. follow) ANOVAs were used for subjective ratings and PFC activity. 2 (age) x 2 (experience) x 2 (gender) x 4 (traffic density) ANOVAs were conducted for subjective ratings, PFC activity and number of overtakes. 2 (age) x 2 (experience) x 2 (gender) x 2 (hemisphere) ANOVAs were used for PFC activity. Finally, 2 (age) x 2 (experience) x 2 (gender) x 3 (pre- during- post- overtake) ANOVAs were conducted for PFC activity for different sections of the overtake manoeuvre. Self-report measures are discussed first, followed by driver behaviour and finally fNIRS data.

## Results

### NASA-TLX: Follow Vs. Overtake

**Fig 3:**
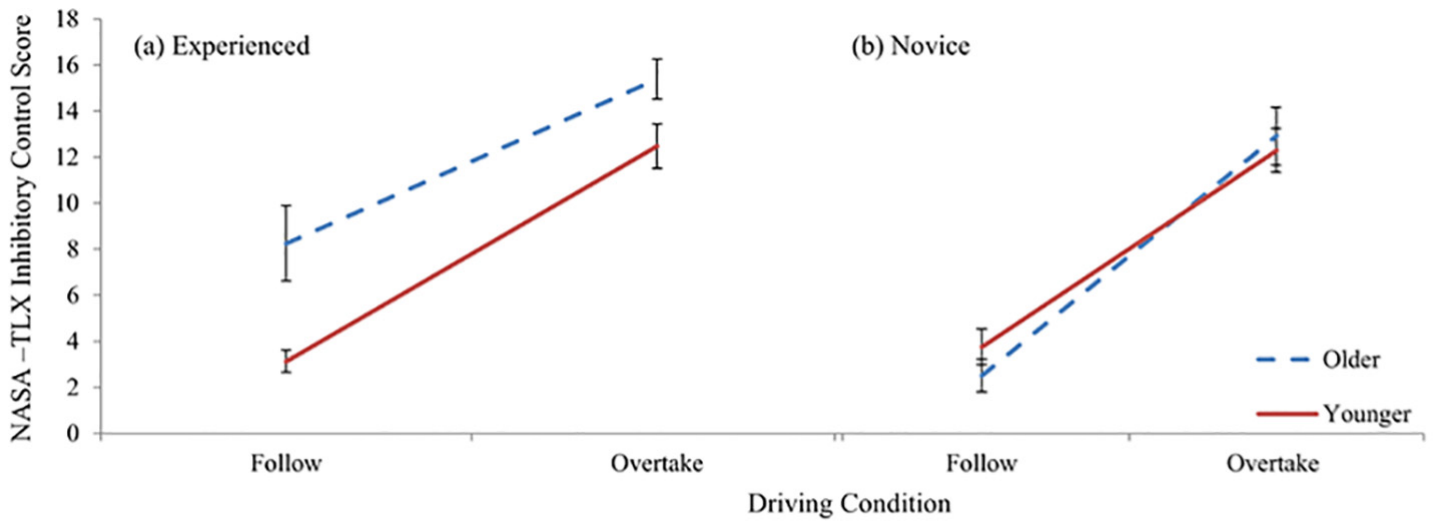
NASA-TLX Inhibitory Control scores. Graph shows age by experience interaction (a) Older experienced drivers reported higher levels of inhibitory control in both the following and overtaking conditions when compared to younger experienced drivers. (b) No differences between older and younger novice driver ratings of inhibitory control. Error bars represent standard error of the mean.

Raw TLX data scores from 0 to 20 were used for analysis. Raw scores were used as they are more sensitive than other methods of data treatment such as scale weighting [68]. ANOVAs showed a main effect of task for Mental Demand (*F*(1,24) = 153.102, *p*<.001, Ƞ_p_^2^ = .864) Participants rated overtaking (*M*: 11.27, *SD*: 3.53) more mentally demanding than following (*M*: 4.44, *SD*: 2.31). Inhibitory Control showed a significant main effect of task (*F*(1,24) = 179.445, *p* <.001, Ƞ_p_^2^ = .882), a significant main effect of age (*F*(1,24) = 5.000, *p* = .035, Ƞ_p_^2^ = .172) and a significant interaction between age group and experience (*F*(1,24) = 6.830, *p* = .027, Ƞ_p_^2^ = .187). Overtaking (*M*: 13.17, *SD*: 3.02) was considered to require more inhibitory control than following (*M*: 4.41, *SD*: 3.54) and the older (Follow *M*: 5.38, *SD*: 1.14, Overtake: *M*: 13.97, *SD*: 0.82) group rated both conditions higher than younger drivers (Follow *M*: 3.44, *SD*: 0.45, Overtake: *M*: 12.38, *SD*: 0.65) in terms of the amount of inhibitory control elicited. Bonferroni corrected simple main effects analysis revealed a main effect of age for experienced drivers (*F*(1,24) = 11.759, *p* = .002, Ƞ_p_^2^ = .033). Older experienced drivers (Follow *M*: 8.25, *SD*: 1.63, Overtake: *M*: 15.38, *SD*: 0.86) reported using higher levels inhibitory control than younger experienced drivers (Follow *M*: 3.13, *SD*: 0.48, Overtake: *M*: 12.47, *SD*: 0.96). No main effect of age was found for novice drivers (Fig 3).

### Increasing Traffic Density

**Fig 4:**
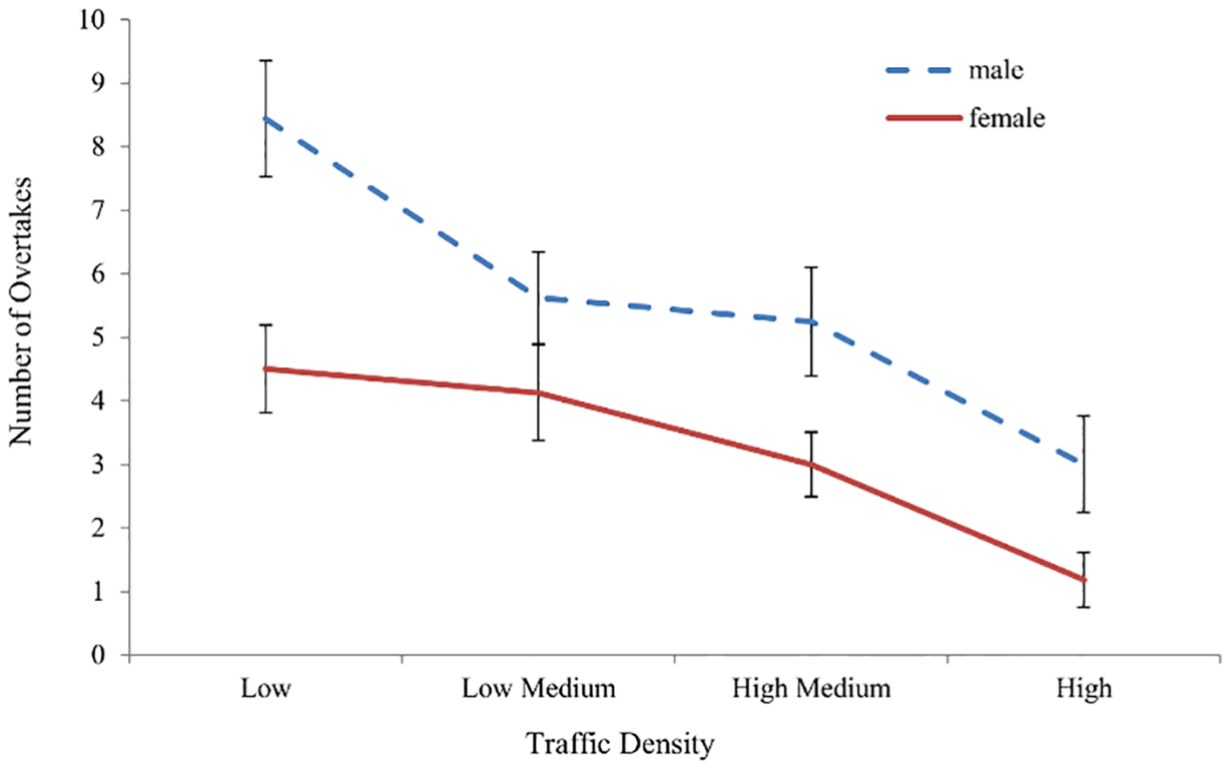
Number of completed overtakes. Graph shows a main effect of task, a main effect of gender and a significant interaction between traffic density (task) and gender. As traffic density increased number of overtakes decreased. Males overtook more than females. This was significant in the low and high medium traffic densities. Error bars represent standard error of the mean.

ANOVAs showed a main effect of task (*F*(3,72) = 35.694, *p* <.001, Ƞ_p_^2^ = .598) with a significant linear trend (*F*(1,24) = 71.083, *p* <.001, Ƞ_p_^2^ = .748). As the traffic density on the opposite side of the carriageway increased the number of overtakes decreased (Fig 4). A main effect of gender (*F*(1,24) = 6.475, *p* = .018,Ƞ_p_^2^ = .212), and a significant interaction between task and gender was also found (*F*(3,72) = 3.190, *p* = .029, Ƞ_p_^2^ = .117). Male participants (*M*: 5.57, *SD*: 3.74) completed more overtakes than females (*M*: 3.20, *SD*: 2.71) across the conditions, simple main effects analysis revealed that this difference was significant in the Low Traffic Density (*F*(1,96) = 13.500, *p* <.001, Ƞ_p_^2^ = .123) and in the High Medium condition (*F*(1,96) = 4.408, *p* = .038, Ƞ_p_^2^ = .044) but was not significant for the Low Medium and High traffic densities. No significant main effect of age was found (*F*(1,24) = .091, *ns* .76).

For the NASA-TLX workload scores, ANOVAs showed no significant main effect for Mental Demand. However, inhibitory control showed a main effect of task (*F*(3,72) = 3.080, *p* = .033, Ƞ_p_^2^ = .114) in a linear direction (*F*(1,24) = 5.994, *p* = .022, Ƞ_p_^2^ = .200) with participants recording that they had to use greater inhibitory control as traffic density increased.

### fNIRS data

Due to technical faults with the fNIRS device no data was recorded for the high density condition for participant 4 and the low and low medium conditions for participants 17 and 27. fNIRS analyses compared averages over the 16 recording channels except where hemisphere analyses were conducted. For hemisphere analyses averages of channels 1 to 8 were used for the left hemisphere and 9 to 16 for the right hemisphere. Where averages are calculated over traffic density the remaining conditions were used. Missing data is represented with blank cells in S1 Appendix.

**Fig 5:**
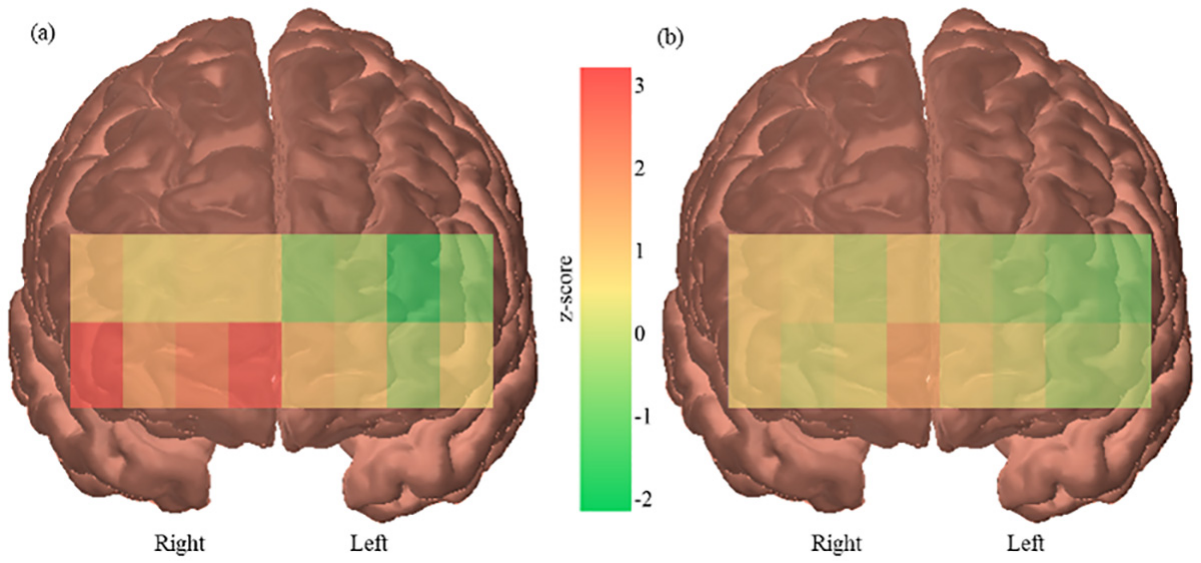
PFC activation during overtaking and following tasks. Channel by channel activation maps showing greater activity in the PFC during overtaking tasks (a) than following tasks (b). Z-scores represent change from resting baseline.

**Fig 6:**
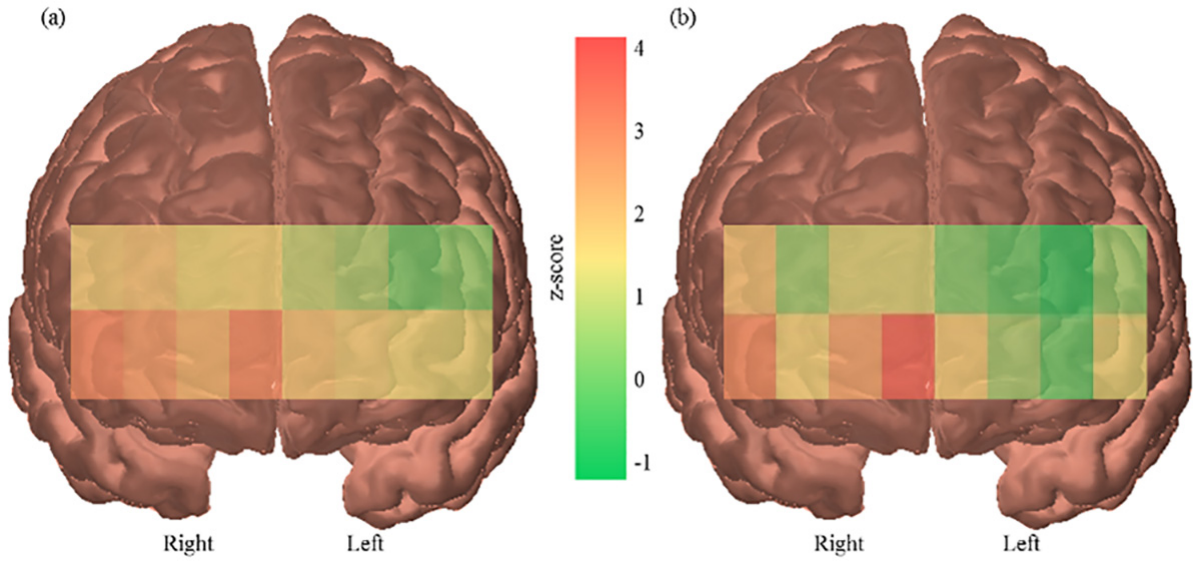
PFC activation of younger and older drivers. Channel by channel activation maps showing greater activity in older drivers (a) than younger drivers (b). Z-scores represent change from resting baseline.

ANOVAs showed a significant main effect of task (*F*(1,24) = 4.481, *p* = .045, Ƞ_p_^2^ = .157) and a significant main effect of age (*F*(1,24) = 5.090, *p* = .033, Ƞ_p_^2^ = .175). Overtaking (*M*: 0.07, *SD*: 0.08) elicited a greater change in PFC activity than following (*M*: 0.04, *SD*: 0.05) (Fig 5) and older drivers (*M*: 0.07, *SD*: 0.08) had a greater increase in PFC activity than younger drivers (*M*: 0.04, *SD*: 0.05) (Fig 6).

There was also a significant task by age by experience by gender interaction (*F*(1,24) = 5.862, *p* = .023, Ƞ_p_^2^ = .196). Bonferroni corrected pairwise comparisons showed that the *a priori* safest group had the greatest activation with old experienced females (*M*: 0.18, *SD*: 0.06) showing greater activity than young experienced females (*p* = .009) (*M*: 0.05, *SD*: 0.08), than old novice females (*p* = .005) (*M*: 0.04, *SD*: 0.03) and old experienced male drivers (*p* = .023) (*M*: 0.07, *SD*: 0.05) in the overtaking condition. Young novice males, the *a priori* most dangerous group had the lowest mean activity (*M*: 0.02, *SD*: 0.05), however, this difference was not significant.

**Fig 7:**
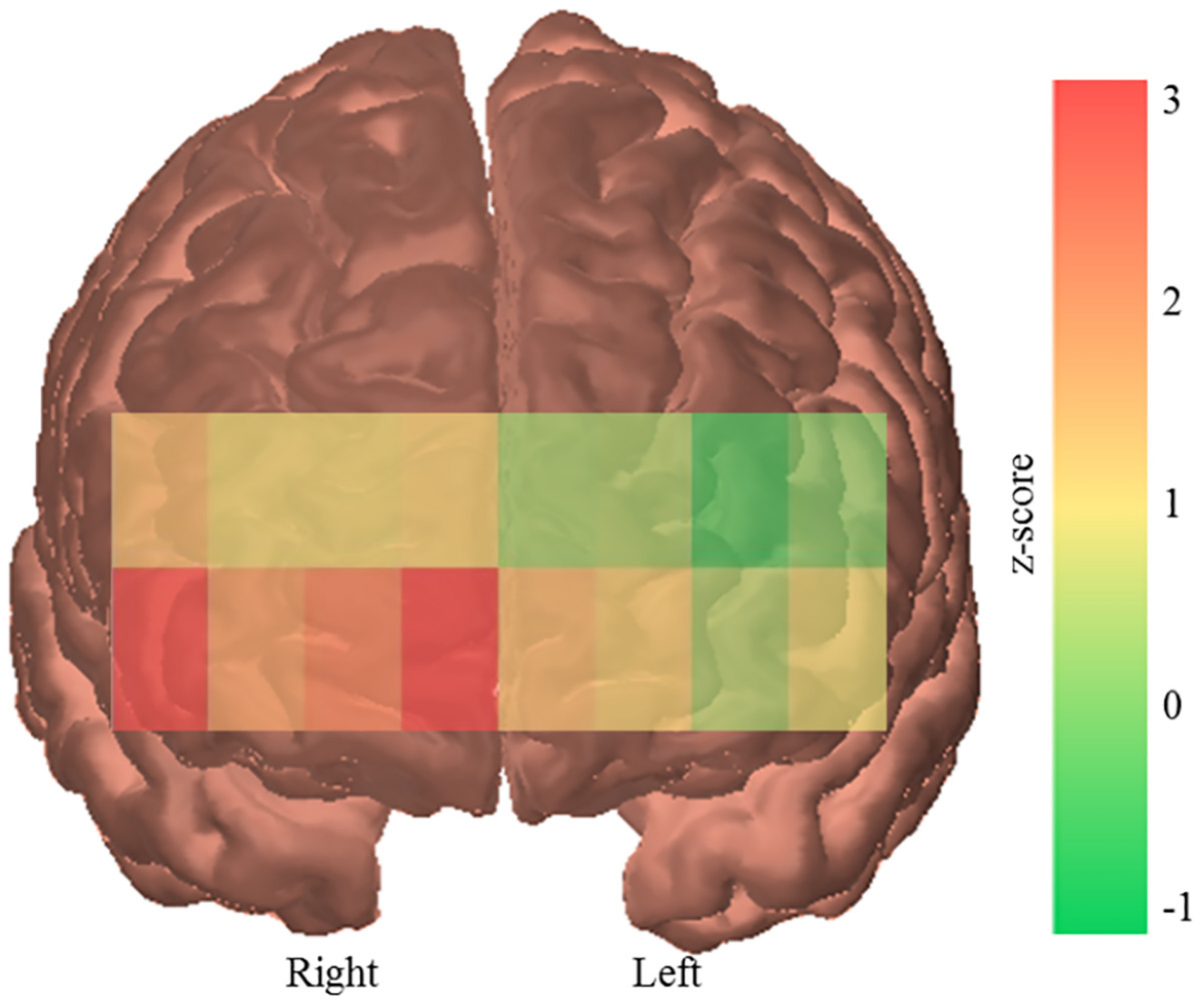
PFC activation by hemisphere. Channel by channel activation map showing greater activity in the right hemisphere of the PFC than the left, measured across all tasks. Z-scores represent change from resting baseline.

ANOVAs also revealed a significant main effect of hemisphere across all tasks (*F*(1,24) = 32.134, *p* <.001, Ƞ_p_^2^ = .572) and for both the overtaking (*F*(1,24) = 31.541, *p* <.001, Ƞ_p_^2^ = .568) and following conditions (*F*(1,24) = 5.978, *p* = .022, Ƞ_p_^2^ = .199) separately. There were greater increases in activity in the right hemisphere (*M*: 0.10, *SD*: 0.09) of the PFC than the left (*M*: 0.02, *SD*: 0.06) (Fig 7).

**Fig 8:**
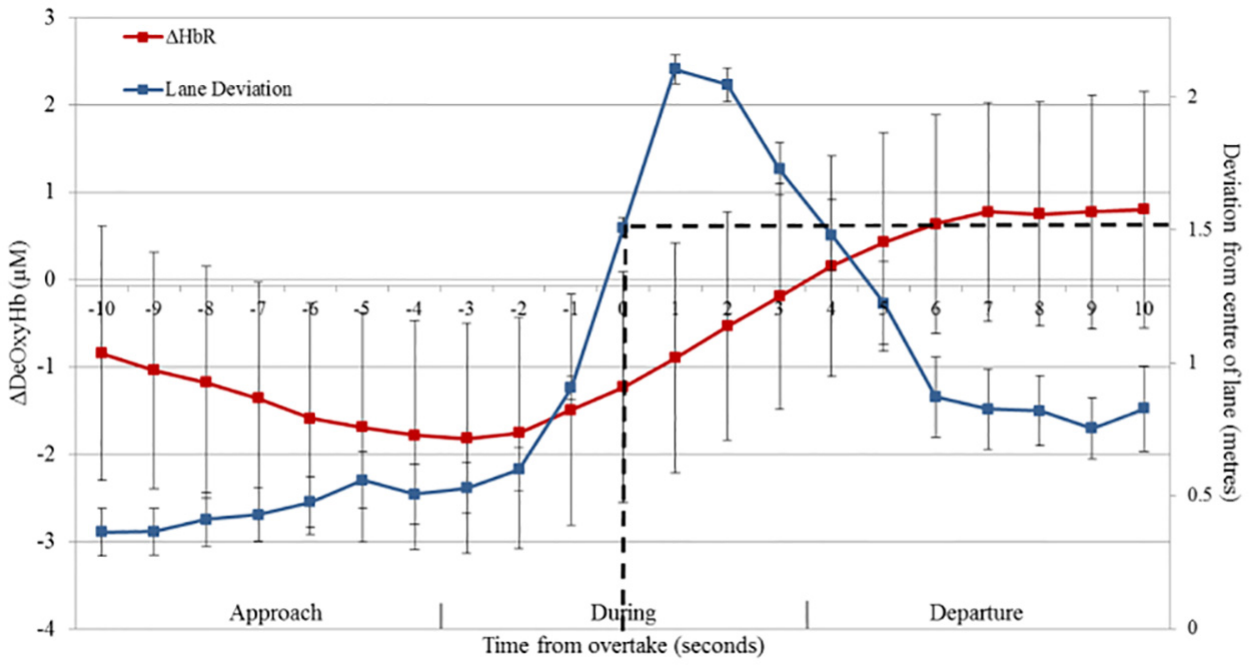
PFC activity changes during overtaking. Graph shows lane deviation and fNIRS data for participants’ first overtake. Results are for ΔDeoxyHb and show a marginally significant main effect of window in a linear direction. The dashed line marks the point at which lane deviation is 1.5 metres from the centre of the lane, this is taken as the point of overtake (0 seconds). From this point approach, during and departure overtake windows of 7 seconds were created. PFC activity increased during the overtake and continued to do so in the overtake departure window. Error bars represent standard error of the mean.

There was no significant difference in PFC activity when comparing the four levels of traffic density (*F*(3,69) = .608, ns .612) in the overtaking conditions. As not all participants overtook in each of the four overtaking tasks (and participant 4 only completed one overtake throughout the experiment) global fNIRS data may not be representative of changes in PFC activity during the specific overtake manoeuvre. Therefore, fNIRS data was extracted for each participant’s first overtake in the Low density condition from 10 seconds before the point of overtake until 10 seconds after. Where the Low density fNIRS data was not available (participants 17 and 27) fNIRS data from the lowest density condition available was used (High Medium). As the order of drives was randomised this method kept the number of measurements taken for each participant consistent whilst controlling for order effects. The point of overtake was defined as the time when vehicle lane deviation was greater than 1.5 metres from the centre of the lane; once participants reached this distance they always overtook. This resulted in 21 seconds of fNIRS data, which was split into three groups of seven seconds to create approach, during and departure overtake windows. ANOVAs indicated a violation of sphericity for this data. Greenhouse-Geisesr corrections revealed a marginally significant main effect of window (*F*(1.42,34.14) = 3.567 *p* = .053, Ƞ_p_^2^ = .129) with a linear trend over time (*F*(1,24) = 3.757, *p* = .064, Ƞ_p_^2^ = .135). PFC activity increased during overtaking and continued to increase into the period immediately after the manoeuvre (Fig 8). There was also a significant quadratic relationship between window and age group (*F*(1,24) = 4.581, *p* = .043, Ƞ_p_^2^ = .160) Older drivers had significantly greater activity in the post overtake window than during the overtake (*p* = .039). There were no significant differences for young drivers.

## Discussion

The aims of this research were to examine the PFC activity of the young driver population and to use simulated driving tasks to manipulate mental workload and inhibitory control; both of which have been linked to young driver accidents and also changes in PFC activity.

As predicted; the older drivers had greater changes in PFC activity than younger drivers. The *a priori* safest driving group (old experienced females) showed the greatest changes in PFC activation and older drivers also showed increased activation following a successful overtake, a manoeuvre which is a high accident risk for young drivers [6]. However, younger drivers showed no such PFC activation changes. As the brain is still developing in this population younger drivers would be expected to have less PFC maturation than older drivers, which, as previous research suggests could account for this reduced activity [24,25,69]. Generally, research has demonstrated a correlation between brain activity and capacity or performance on a task [70,71]. Therefore reduced activation in younger drivers could be associated with poorer driving skill and performance and thus increased road traffic accidents. More specifically for this research; in terms of inhibitory control previous evidence demonstrates increased risk taking is correlated with reduced prefrontal activity [34]. With respect to mental workload, due to their reduced capacity the threshold for overload and errors is lower and as inexperienced drivers are already operating at a higher workload [47] this further reduces the availability for workload increase in young novice drivers.

Both inhibitory control and mental workload may play a role in young novice accidents. However, this research suggests that PFC activity is more indicative of mental workload changes than inhibitory control changes in the current task. This is based on a number of results; overtaking increased both perceived inhibitory control and mental workload when compared to the following task. PFC activity showed the same pattern in that there was greater PFC activity in the overtaking tasks. However, our manipulation of traffic density created a situation in which mental workload remained relatively constant whilst inhibitory control increased. Our intention was to create a situation in which increases in traffic density increased the amount of time in which it was not safe to overtake and thus cause drivers to inhibit their desire to initiate overtaking manoeuvres. The inhibitory control scale suggests that this manipulation was successful. This change in inhibitory control was not reflected in PFC activity as no differences were seen between the different traffic density conditions. Looking more specifically at the time of overtake also suggests that mental workload makes a clearer contribution. As inhibitory control relates to the decision to perform a risky manoeuvre, the associated haemodynamic response would be expected to occur in the approach to the overtake whereas these results demonstrate an increase during the overtake and in the period immediately after. This is more likely associated with the added workload of successfully executing the overtake manoeuvre. Even with a hemodynamic delay of approximately 6 seconds [72,73] the peak of the response would still occur during the overtake, after the decision has been made.

Despite seeing differences in PFC activity associated with age and with changes in mental workload there were no age or experience differences in self report measures of mental workload. Although unexpected this is an interesting result which suggests that younger novice drivers do not feel that overtaking requires a degree of workload beyond their capabilities; the danger with this is that they may also be unaware of their reduced threshold for errors and may subsequently struggle to identify high workload situations where errors occur. This is consistent with evidence that novices overestimate driving skill, underestimate accident risk and have poorer situational awareness [74–76]. As fNIRS did show differences in PFC activity relating to mental workload and age it may be possible to implement fNIRS as a detection tool for different mental workload states [77] and in particular to examine and detect overload situations in young novice drivers.

Contrary to our expectations younger drivers did not overtake more, showing no additional risk taking despite older drivers reporting that they used more inhibitory control. One possibility is that older drivers were safer in when they chose to overtake rather than how often. This could be investigated in future by measuring gap acceptance or indecision time [78]. For example by comparing the time available for the overtake and the time required to complete the overtake. In contrast, male drivers did overtake more than female drivers, as predicted. This supports previous research and statistics that males take more risks when driving [14,17].

Results also showed that there was more activity in the right hemisphere of the PFC (channels 9–16) than the left (channels 1–8). This is consistent with previous research by Shimizu, Nanbu and Sunda (2011) [79] who found that when operating a right hand drive vehicle (as used in the current experiment) there was greater activation in the right hemisphere of the frontal lobe. In contrast, when participants operated a left hand drive vehicle greater activation was observed in the left hemisphere. Results are also consistent with evidence that activity is greater on the side of space contralateral to the allocation of attention [80–83]. As there is an added difficulty and thus workload of perceiving the far side of the vehicle attention must be directed in the opposite direction of the driver’s seat (i.e. the left in this experiment). This is particularly crucial in this experiment as when overtaking care must be taken to avoid colliding with the other vehicle.

Although this experiment suggests that PFC activity is related to some aspects of mental workload during driving there were not consistent workload changes throughout each task. In contrast changes in inhibitory control were seen both when comparing overtaking and following tasks and when examining changes in traffic density. Therefore, a task in which mental workload changes and inhibitory control remains constant should be implemented in future in order to see if each mental workload change is also accompanied by a change in PFC activity. Variations in road type could be used to manipulate mental workload in a naturalistic driving task, for example areas with high traffic density and numerous curves and junctions which have previously been classed as high complexity [84]. These characteristics of high workload road types are typically found in urban areas which are also the environments that demonstrate the greatest accident rates [85].

Although this research achieved its aim of investigating young driver PFC activity and successfully demonstrated that younger drivers have reduced activity it may be beneficial to implement a longitudinal design with structural scans in the future in order to examine changes in functional activity and associated structural development over time. Although the suggestion posed by the current research that reduced activity is related to reduced maturation is supported by previous research [24,25,52], it would be beneficial to provide direct evidence of this association. Furthermore, previous research has suggested that NIRS could be used to assess brain development [52].

It may also be advantageous to examine other populations in future. For example the brain reaches maturation around age 25 [28] and so we would expect drivers of this age to also have increased activity compared to teenagers and their slightly younger (age 21) counterparts. It would also be valuable to examine older populations (age 55+) as this age group also have a higher rate of fatal crashes [86] and evidence suggests that brain size begins to reduce at approximately 40 years of age [87,88]. More specifically, studies have shown age related volumetric reductions in prefrontal regions [89]. Furthermore, research has demonstrated decreases in PFC activity for older participants (over 55) which were accompanied by deficits in performance as a result of increases in workload demands [89]. Thus, reductions in brain size and associated cognitive slowing [90] in older drivers may also contribute to the reduced performance and increased crash risk seen for these drivers and may be evident in their PFC activity. However, a major consideration when examining older populations is that as age increases it becomes more difficult to successfully manipulate experience levels.

In conclusion, the results of this study support fNIRS as a valuable neuroimaging technique which can be used in realistic situations such as vehicle driving and could be implemented in the assessment and prediction of driver overload and subsequent error. Both mental workload and inhibitory control have been linked to road traffic collisions and PFC activity, however PFC activity appears to be more indicative of mental workload changes particularly in the current simulated driving scenario. Finally, younger drivers, even within the confines of a relatively young driver sample showed significantly less PFC activity than the older drivers. As the PFC is still maturing during this phase this could go some way to explain the high accident and fatality risk in this population.

## Supporting Information

S1 Appendix Data for NASA-TLX, Traffic Density and fNIRS analysis. (XLSX)
